# Development and Validation of a Real-Time PCR for *Chlamydia suis* Diagnosis in Swine and Humans

**DOI:** 10.1371/journal.pone.0096704

**Published:** 2014-05-09

**Authors:** Kristien De Puysseleyr, Leentje De Puysseleyr, Julie Geldhof, Eric Cox, Daisy Vanrompay

**Affiliations:** 1 Department of Molecular Biotechnology, Faculty of Bioscience Engineering, University of Ghent, Ghent, Belgium; 2 Department of Virology, Parasitology and Immunology, Faculty of Veterinary Medicine, University of Ghent, Ghent, Belgium; University of Lausanne, Switzerland

## Abstract

Pigs are the natural host for *Chlamydia suis*, a pathogen which is phylogenetically highly related to the human pathogen *C. trachomatis*. *Chlamydia suis* infections are generally treated with tetracyclines. In 1998, tetracyline resistant *C. suis* strains emerged on U.S. pig farms and they are currently present in the Belgian, Cypriote, German, Israeli, Italian and Swiss pig industry. Infections with tetracycline resistant *C. suis* strains are mainly associated with severe reproductive failure leading to marked economical loss. We developed a sensitive and specific TaqMan probe-based *C. suis* real-time PCR for examining clinical samples of both pigs and humans. The analytical sensitivity of the real-time PCR is 10 rDNA copies/reaction without cross-amplifying DNA of other *Chlamydia* species. The PCR was successfully validated using conjunctival, pharyngeal and stool samples of slaughterhouse employees, as well as porcine samples from two farms with evidence of reproductive failure and one farm without clinical disease. *Chlamydia suis* was only detected in diseased pigs and in the eyes of humans. Positive humans had no clinical complaints. PCR results were confirmed by culture in McCoy cells. In addition, *Chlamydia suis* isolates were also examined by the *tet*(C) PCR, designed for demonstrating the tetracycline resistance gene *tet*(C). The *tet*(C) gene was only present in porcine *C. suis* isolates.

## Introduction

*Chlamydiaceae* are obligate intracellular Gram-negative bacteria that cause a broad range of diseases in both humans and animals. Pigs can become infected by *Chlamydia (C.) pecorum*, *C. abortus*, *C. psittaci* and *C. suis* [1]. Seroprevalence rates of *Chlamydiaceae* in German, Swiss, Italian and Belgian pigs are high (40–96%) [2], [3], [4], [5]. As far as known, the pig is the only host for *C. suis,* which is phylogenetically closely related to the human pathogenic agent *C. trachomatis* [1]. *Chlamydia suis* infections can be asymptomatic or associated with arthritis, pericarditis, polyserositis, pneumonia, conjunctivitis, enteritis, diarrhea and reproductive failure [4], [6], [7], [8], [9], [10]. Experimental infections in specific pathogen free and gnotobiotic pigs using *C. suis* field strains have resulted in conjunctivitis, respiratory lesions and gastrointestinal lesions [11], [12], [13]. To date, transmission of *C. suis* to humans has not been reported [14]. However, *C. suis* and *C. trachomatis* are closely related [1] and, therefore, the zoonotic potential of *C. suis* is likely.

*Chlamydia suis* can be difficult to grow in cell culture or embryonated eggs [14]. A molecular diagnostic test, able to specifically detect *C. suis* in both animal and human samples, is crucial to gain insights into the prevalence, the economic impact and the zoonotic ‘threat’ of *C. suis* infections. In 2006, Sachse et al. [15], developed a 23S rDNA gene-based array tube (AT) micro array for detection and differentiation of *Chlamydia* spp. The assay detects a single copy of the cloned target DNA [16]. Borel *et al*. [17] calculated the sensitivity of the AT micro array over an entire panel of human and animal clinical samples, including five human pharyngeal swabs and four tissues of naturally infected diseased pigs. The sensitivity of the AT micro array was slightly lower than for real-time PCR. When excluding long-time stored swab samples from the calculation, the sensitivity was equivalent to that of real-time PCR. However, in our hands, *C. suis* species identification by use of the AT microarray was often only possible on isolates of vaginal and rectal swabs and not directly on these clinical samples [18]. More recently, Pantchev et al., [19] developed a 23S rDNA gene-based real-time PCR for *C. suis*. However, the PCR was strictly developed for the examination of veterinary samples as it cannot distinguish *C. suis* from the genetically closely related human pathogen *C. trachomatis* [19]. Therefore, it was the aim of the present study to develop a sensitive and specific *C. suis* real-time PCR for the purpose of examining clinical samples of both pigs and humans.

## Materials and Methods

### 1. Bacterial cultures

For determination of sensitivity, specificity and detection limit of the *C. suis*-specific real-time PCR, the following 14 chlamydial strains were used: *C. suis* strains S45, R19, H7, R24, R27 and R33, *C. pneumoniae* (TW-183), *C. felis* (FP Baker), *C. caviae* (GIPC), *C. abortus* (S26/3), *C. psittaci* (92/1293), *C. pecorum* (1710S), *C. muridarum* (MoPn) and *C. trachomatis* (L2/434/BU). Bacteria were grown in cycloheximide treated McCoy cells (mouse fibroblast cells, obtained from Els De Coster, Labo Medische Analyse, CRI, Zwijnaarde, Belgium; original source: ATCC CRL-1696) using a standard methodology, as described previously [20]. Chlamydiae were released from infected monolayers (two passages) by freezing and thawing, followed by ultrasonication (Bransonic 12, BIOMEDevice, San Pablo, CA, USA). Cell culture harvest was centrifuged for 10 min (1000 × *g*, 4°C) and *Chlamydiae* were subsequently concentrated by ultracentrifugation for 45 minutes (50,000 × *g*, 4°C). Bacteria were resuspended in 2 mL sucrose phosphate glutamate buffer (SPG, 218 mM sucrose, 38 mM KH_2_PO_4_, 7 mM K_2_HPO_4_, 5 mM L-glutamic acid) and stored at –80°C until use.

### 2. DNA extraction

Genomic DNA of bacterial cultures was prepared using the DNeasy Blood and Tissue Kit (Qiagen, Antwerp, Belgium). DNA extraction of clinical samples was performed using the G-spin Total DNA Extraction Mini Kit (Goffin Molecular Biotechnologies, Beek, The Netherlands). Both extraction methods were performed according to the instructions of the manufacturers.

### 3. Primers and probes

Published 23S ribosomal RNA sequences of all known chlamydial species (Table 1) [1] were aligned using Clustal X software (default settings) [21]. Forward and reverse primers (Life technologies, Paisley, United Kingdom) and probes (Eurogentec, Seraing, Liège, Belgium) were designed using primer3 software (Table 2) [22]. Primer and probe specificity was checked by BLAST [21]. The optimal annealing temperature and specificity of the primers and probes within the genome was evaluated in 20 µl PCR reactions containing 3 µl of 2 µM of forward and reverse primers, 0.5 µl Super Taq 5U/µl (Sphaero Q, Gorinchem, The Netherlands), 2 µl Super Taq Reaction Buffer, 2 µl of each of 1.25 mM dNTP and 50 ng of genomic DNA of *C. suis* strain S45. The cycling conditions were as follows: 72°C for 5 minutes; 35 cycles of 95°C for 60s, 60°C for 60s and 72°C for 60s and a final step of 72°C for 5 min. CS23S-probe A and B were 5′ labeled with the reporter dye 6-carboxyfluorescein (FAM) and hexachloro-fluoresceine (HEX) respectively, and 3′ labeled with the quencher dye carboxytetramethylrhodamine (TAMRA).

**Table 1:** NCBI Nucleotide Accession numbers of the 23S rRNA gene sequences that were used for the alignment to design the primers and *C. suis* specific probes.

Species	Strain	NCBI Nucleotide Accession Number
*Chlamydia suis*	R22	U68420
*Chlamydia trachomatis*	L2/434/BU	U68443
*Chlamydia abortus*	EBA	U76710
*Chlamydia psittaci*	NJ1	U68419
	6BC	U68447
*Chlamydia pecorum*	IPA	U68434
*Chlamydia felis*	FP Baker	U68457
*Chlamydia muridarum*	MoPn	U68436
	SFPD	U68437
*Chlamydia caviae*	GPIC	U68451
*Chlamydia pneumoniae*	TW-183	U76711

**Table 2:** Primers and probes of the *C. suis* specific real-time PCR.

Oligonoculeotide	Sequence (5′- 3′)	Position^a^	Specificity
CS23S-F	GCAGAGGAAAAGAAATCGAAGA	215–236	*C. suis and C. trachomatis*
CS23S-R	CGGGACTATCACCCTGTATC	359–378	*C. suis and C. trachomatis*
CS23S-ProbeA	FAM-CGAGCTGAAGAAGCGAGGGGTTGTAG-TAMRA	280–305	*C. suis*
CS23S-ProbeB	HEX-CGAGCCGAAGAAGCGAGGGGTTGTAG-TAMRA	280–305	*C. suis*

a Binding position from base 1 of the 23S rRNA gene of the chlamydial reference strain S45.

### 4. Inhibition control plasmid

The 23S rDNA amplicon of *C. suis* (S45) was amplified using 20 µl PCR reactions containing 3 µl of 2 µM of forward and reverse primers, 0.5 µl Super Taq 5U/µl (Sphaero Q, Gorinchem, The Netherlands), 2 µl Super Taq Reaction Buffer, 2 µl of each of 1.25 mM dNTP and 50 ng of genomic DNA. PCR products were purified using the Wizard SV Gel and PCR Clean-Up System (Promega, Madison, WI, USA) and cloned into pGem-T (Promega, Madison, WI, USA) according to the manufacturer's protocol. To verify the nucleotide sequence of the inserted fragment, sequence analyses were performed by the VIB Genetic Service Facility (University of Antwerp, Antwerp, Belgium) using vector associated T7 and SP6 priming sites. The resulting *C. suis-*species-specific internal inhibition control plasmid was designated pGemT::CSIC.

### 5. Species-specific real-time PCR

Real-time PCR was performed with the Rotor-Gene Q Instrument (Qiagen Benelux, Venlo, The Netherlands) using 25 µl reaction mixture containing 2 µl of DNA template, 4 µl of primer mixture (300 nM forward and reverse primer), 2.5 µl of CS23S-probe A and B (200 nM), 12.5 µl of absolute qPCR mix (Thermoscientific, Acros Organics, Geel, Belgium) and 1.5 µl DNAse and RNAse free water. The cycling conditions were as follows: 95°C for 15 minutes, 50 cycles of 95°C for 15 seconds, 60°C for 60 seconds. All default program settings were used. Standard graphs of the Cycle threshold (Ct) values, obtained by testing tenfold serial dilutions (10^8^ to 10^1^) of the purified species-specific inhibition control plasmid, were used for quantification. DNA was always tested in the presence of control plasmid (50 copies/µl) to check for PCR inhibitors.

### 6. Analytical sensitivity and specificity

The analytical sensitivity of the assay was evaluated using decimal serial dilutions (10^8^ to 10^1^ copies/µl) of the inhibition control plasmid and of purified genomic DNA of the *C. suis* reference strain S45 from suspensions equivalent to 10^8^ to 10^1^ copies/µl. Three independent dilution series were analyzed and reactions were performed in triplicate. The specificity was evaluated using: i) genomic DNA from *C. peumoniae (TWAR)*, *C. felis (FP Baker)*, *C. caviae (GPIC)*, *C. abortus(S26/3)*, *C. psittaci (92/1293)*, *C. pecorum (1710S)*, *C. muridarum (MoPn)* and *C. trachomatis (*L2/434/BU*)* as well as ii) DNA from 23 and 38 bacterial species commonly found in swine and humans, respectively, and iii) genomic DNA from swine and human tissues (Table 3 and 4).

**Table 3:** Organisms of non-chlamydial origin found in swine and used for specificity testing.

Organisms*		
*Acinetobacter calcoaceticus*	*Enterobacter sp.*	*Pseudomonas aeruginosa*
*Bacteroides sp.*	*Enterococcus sp*	*Proteus mirabilis*
*Bordetella bronchiseptica*	*Escherichia coli*	*Proteus vulgaris*
*Bordetella pertussis*	*Klebsiella sp.*	*Salmonella enterica subsp. enterica serovar Typhimurium*
*Bordetella parapertussis*	*Lactobacillus sp.*	*Staphylococcus aureus*
*Brachyspira sp.*		*Staphylococcus hyicus*
*Brucella sp.*	*Mycoplasma hyopneumoniae*	*Streptococcus suis*
*Clostridium sp.*	*Pasteurella multocida*	*Treponema sp.*

* Identification of micro-organisms was performed by culture on selective media, biochemical identification and molecular characterization methods like PCR and DNA sequencing.

**Table 4:** Organisms of non-chlamydial origin found in humans and used for specificity testing.

Organisms*		
*Acinetobacter baumannii*	*Klebsiella oxytoca*	*Ruminococcus*
*Bacteroides sp.*	*Klebsiella pneumoniae*	*Salmonella sp.*
*Bifidobacterium sp.*	*Lactobacillus sp.*	*Serratia marcescens*
*Bordetella bronchiseptica*	*Stenotrophomonas sp*	*Shigella sp.*
*Citrobacter braakii*	*Moraxella catarrhalis*	*Staphylococcus aureus*
*Citrobacter freundii*	*Morganella morganii*	*Staphylococcus epidermidis*
*Clostridium sp*	*Mycoplasma pneumoniae*	*Moraxella catarrhalis*
*Corynebacterium sp.*	*Peptostreptococcus sp.*	*Streptococcus agalactiae*
*Enterobacter sp.*	*Streptococcus pneumoniae sp.*	*Streptococcus mitis*
*Enterococcus sp.*	*Propionibacterium sp.*	*Streptococcus nilleri*
*Escherichia coli*	*Pseudomonas aeruginosa*	*Streptococcus pyogenes*
*Eubacterium sp.*	*Proteus mirabilis*	*Veillonella sp.*
*Faecalibacterium sp.*	*Proteus vulgaris*	

* Identification of micro-organisms was performed by culture on selective media, biochemical identification and molecular characterization methods like PCR and DNA sequencing.

### 7. Case studies

#### 7.1. Pig farms

To validate the performance of the *C. suis* real-time PCR, vaginal swabs originating of 10 to 15 sows from three Dutch pig (A, B, C) farms were tested. Two of the three farms were dealing with cases of reproductive failure (decrease of the conception rate from 90 to <65%, 1–3% abortions, sows that delivered only two to five piglets of a non-uniform weight, white to yellow non-smelling vulval liquid, irregular return to estrus). Clinical disease did not improve following medication with doxycycline [400 g (ton feed) ^−1^] plus a combination of trimethoprim [120 g (ton feed) ^−1^] and sulfamethoxazole [600 g (ton feed) ^−1^] for 10–14 days during gestation and half of these drug concentrations during lactation. Antibody detection or PCR for *Leptospira* species, *Mycoplasma* species, *Brucella suis*, porcine reproductive and respiratory syndrome virus, Aujeszky's disease, swine influenza, porcine circovirus 2 and porcine parvovirus, were negative. Tests were performed at the Dutch Animal Health Service (Deventer, The Netherlands) and by the Flemish Animal Health Service (Drongen, Belgium). The third farm reported no clinical problems and was included as a negative control.

Animals were sampled for *C. suis* culture and PCR. For culture, rayon-tipped aluminium-shafted swabs (Copan; Fiers, Kuurne, Belgium) were immersed in chlamydia transport medium [20] immediately after vaginal sampling and stored at −80°C for culture. For PCR, vaginal swabs immersed in DNA/RNA stabilization buffer (Roche) were stored at −80°C. All DNA extractions were performed using the G-spin Total DNA Extraction Mini Kit (Goffin Molecular Biotechnologies, Beek, The Netherlands) according to the manufacturer‚s protocol.

Purified DNA of vaginal swabs was analyzed by the newly developed *C. suis*-specific real-time PCR. Presence of viable *Chlamydiae* was determined by inoculation on McCoy cells (in duplicate) followed by direct immunofluorescence (anti-LPS/FITC) staining on the first culture drum at six days post inoculation (Imagen, Oxoid, Drongen, Belgium). If positive, the second culture drum was used for *C. suis* identification by use of the newly developed real-time PCR. Positively identified *C. suis* isolates were also examined by the *tet*(C) PCR, designed for demonstrating the tetracycline resistance gene *tet*(C) [23], For this purpose, we used genomic DNA of the Tc^R^ *C. suis* strain R19 [24] as positive control.

All animal samples were received from veterinary practitioners and were delivered for diagnostic purpose. Therefore, approval of the veterinary ethical committee was not required.

#### 7.2. Slaughterhouse employees

Validation of the *C. suis* PCR for human diagnosis was performed by sampling abattoir workers. During a yearly medical check up, employees of a Belgian pig slaughterhouse were asked (informed consent) to voluntarily provide a pharyngeal and conjunctival swab, as well as a swab of fresh stool. Rayon-tipped aluminium-shafted swabs were immersed in chlamydia transport medium [20] and stored at −80°C until culture. Swabs for PCR were immersed in DNA/RNA stabilization buffer and stored at −80°C. Purified DNA was analyzed by the newly developed *C. suis*-specific real-time PCR. Presence of viable *Chlamydiae*, identification of viable *C. suis* and the presence of the *tet*(C) gene in *C. suis* strains was examined as mentioned for the pig farms.

The case study was approved by the medical ethical committee (approval EC UZG 2011/459). Participants provided their written informed consent and the consent procedure was approved by the medical ethical committee of Ghent University.

## Results

### 1. Primers and probes

It was impossible to design *C.°suis*-specific primers in the 23S rDNA target region, such that the forward and reverse primers would also not anneal to *C.°trachomatis* DNA. However, we were able to design *C. suis*-specific probes for real-time PCR. The differentially labeled probes CS23S-A and CS23S-B were *C. suis*-specific and they were both required for coverage of the selected target region of all known *C. suis* strains (Table 2). Forward (CS23S-F) and reverse (CS23S-R) primers generated a PCR product of 159 bp.

### 2. Internal inhibition control

**Figure 1:**
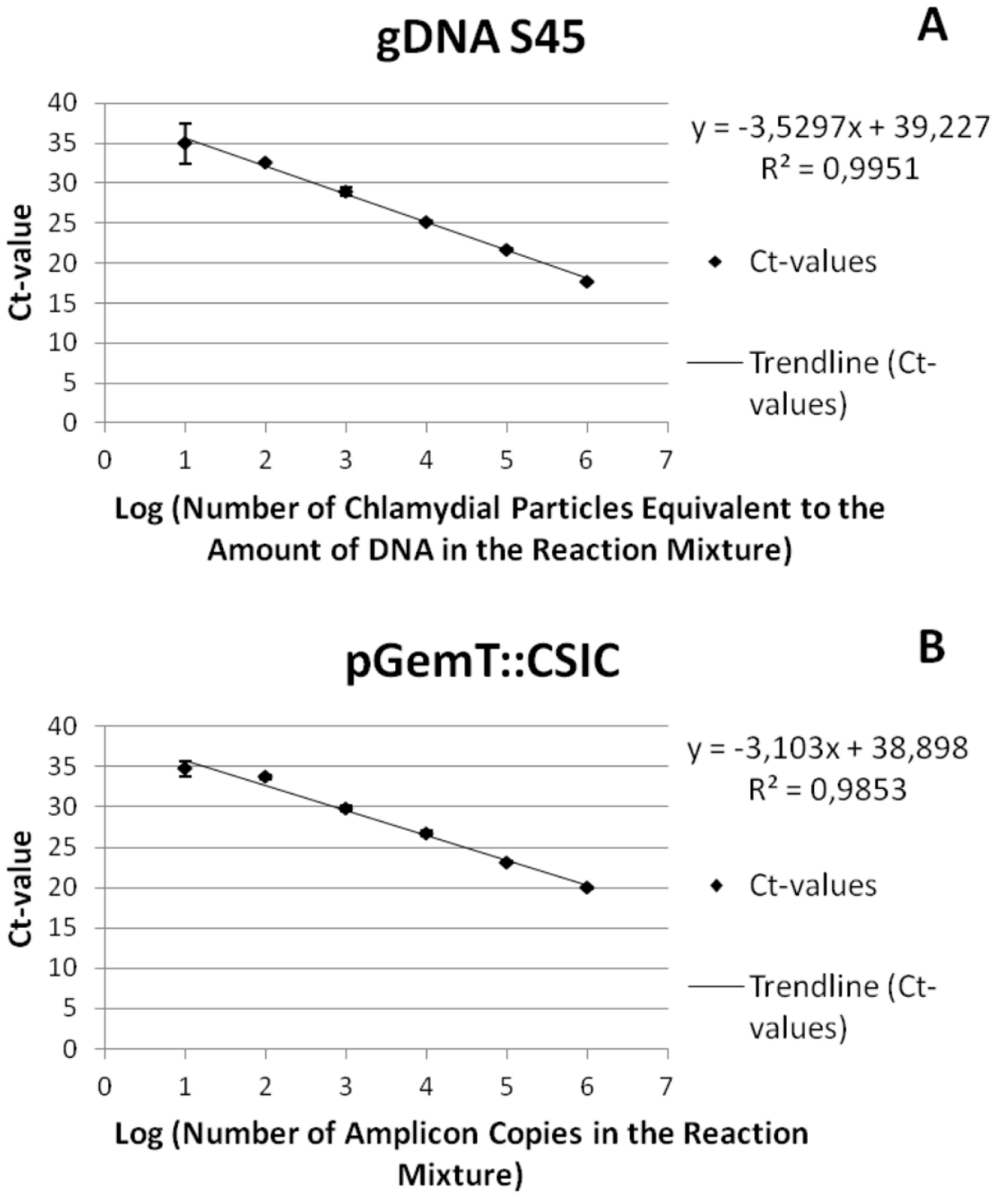
Real-time PCR quantitation of chlamydial S45 genomic DNA (A) and pGemT::CSIC control plasmid (B). The standard curve data points are the average of 3 replications, standard deviations are shown as error bars. The equations and R^2^ linearity values resulting from the linear regression analysis, are shown on the graphs.

Standard curves made using 10^6^ to 10^1^ copies of the pGemT::CSIC control plasmid (Figure 1) showed slopes around −3.103, which correlates to an efficiency of 109%, with correlation coefficients > 98%.

### 3. Species-specific real-time PCR

The species-specific real-time PCR detected all known *C. suis* strains. Amplification of genomic DNA extracts of all *C. suis* strains resulted in the expected PCR products with amplification curves exceeding the 0.02 threshold before cycle 25. The species-specific PCR was able to detect 10 rDNA copies (or two copies/µl) of both genomic and control plasmid DNA. Standard curves resulting from the sensitivity analysis are shown in Figure 1. The slopes were around −3.5, which correlates to an efficiency of 91%, with correlation coefficients > 99%. The species-specificity test revealed a Ct-value of 19 for DNA of 10^6^ TCID_50_ *C. suis.* Real-time PCR on genomic DNA of *C. pneumoniae*, *C. felis*, *C. caviae*, *C. pecorum*, *C. abortus*, *C. muridarum* and *C. trachomatis* generated no signal. The real-time PCR gave no results for genomic DNA of other pathogens commonly found in the eyes, respiratory or urogenital tract of swine and humans, neither with DNA from swine or human tissues.

### 4. Case studies

#### 4.1. Pig farms

*Chlamydia suis* was not detected on the clinically healthy farm C. The pathogen was present on farms A and B, both dealing with reproductive failure. *Chlamydia suis* DNA was found in all (100%) vaginal swabs of farms A and B. Viable *C. suis* organisms were present in 7 of 10 (70%) and 11 of 15 (73.3%) vaginal swabs of farms A and B, respectively. Chlamydial isolates of farm A and B were examined for the presence of the *tet*(C) gene. The *tet*(C) gene was discovered in two of seven (28.5%) and 6 of 11 (54.5%) *C. suis* isolates of farms A and B, respectively. Clinical symptoms disappeared after treatment with enrofloxacin (fluoroquinolone, Baytril 5%; Bayer Healthcare), as previously described [25]. Enrofloxacin was added to the sperm diluter [2 ml (l diluter) ^−1^] and it was also used to rinse the sow's reproductive tract (Baytril 5%, 5 ml+95 ml distilled water) immediately before artificial insemination. Infected sows were re-examined two weeks after treatment and were negative in both PCR and culture.

#### 4.2. Slaughterhouse employees

Only 12 of 84 (14.3%) employees voluntarily participated. They provided 12 conjunctival, 12 pharyngeal and 12 stool swabs. Pharyngeal swabs and stool were all *C. suis* negative. Two of 12 (16.6%) conjunctival swabs were positive by the newly developed *C. suis* real-time PCR, revealing a Ct-value of 26 and 28, respectively. Real-time PCR results on all swabs could be confirmed by culture. Culture harvest of both positively identified *C. suis* isolates was negative by the *tet(C)* PCR. Positive employees worked daily for three years in the pig gut washing area and for eight years in the animal reception area, respectively. Clinical signs were absent.

## Discussion

Chlamydial infections are treated with tetracyclines. In 1998, tetracyline resistant (Tc^R^) *C. suis* strains emerged in the U.S. pig industry (Iowa and Nebraska), and are currently also known to be prevalent in the Belgian, Cypriote, German, Israeli, Italian and Swiss pig industry [2], [3], [9], [26], [27]. The present study adds an additional country to the list, as the current study is the first to discover Tc^R^ *C. suis* strains in the Dutch pig industry. The *C. suis* Tc^R^ phenotype is manifested through the tetracycline resistance gene *tet*(C). *Tet*(C) is integrated into the chlamydial chromosome [28], but it is a transposable genetic element, which is also present in other bacteria such as *E. coli*.

The international economic consequences of *C. suis* infections are not yet fully established, but the financial loss due to severe reproductive failure and the need for antibiotic treatment currently worries pig producers all over the world. Moreover, pig farmers are aware of the existence of Tc^R^ *C. suis* strains and the risk of importing Tc^R^ *C. suis* contaminated sperm for artificial insemination [18] (and P. Delava, personal communication 2014). Furthermore, emergence of Tc^R^ chlamydial strains might pose a potential threat for public health if this species turns out to be a zoonotic pathogen. Suchland *et al*. demonstrated *in vitro* horizontal transfer of the tetracycline resistance gene among chlamydial species [29]. Therefore, contact between Tc^R^ and Tc^S^ *Chlamydia* spp. could lead to transfer of the *tet(C)* gene and the subsequent phenotype, which could then be propagated and selected for in patients that are treated with tetracyclines. However, the lack of evidence of antibiotic resistance leading to treatment failure in humans, seems to indicate that horizontal transfer of the *tet(C)* gene is rather rare. Sandoz and Rockey [30] state that unsufficiency of the current diagnostic methods, could be the cause of this lack of evidence.

In this respect, studying the presence of *C. suis* in humans, dealing with pigs on a regular basis, is meaningful. Thus, there is an urgent need for a highly sensitive and specific molecular diagnostic test to study the epidemiology of *C. suis* in pigs and to study a possible transfer of *C. suis* to humans.

Real-time PCR technology offers the possibility to automatically combine amplification, specific hybridization and detection in one single test. It allows specific and sensitive gene quantification with a minimal contamination risk, as the AmpErase UNG system can be incorporated in order to prevent post-PCR carry-over of amplified DNA. The technique has been used successfully to detect C. *pneumoniae* [31], [32], [33], [34], C. *trachomatis* [35], C. *felis* [36], [37], [38], C. *pecorum* [39], C. *psittaci* [40], and *C. abortus* [19].

Although, the high genomic sequence similarity between *C. suis* and *C. trachomatis* made it difficult to design a sensitive *C. suis*-specific PCR, the analytical sensitivity of the assay was 10 rDNA copies/reaction (or two copies/µl). Besides, the *C. suis* real-time PCR contains two differentially labeled probes. Albeit differentially labeling is not required for the detection of all *C. suis* strains, their use creates the ability to differentiate the two ‘groups’ of *C. suis* strains which are distinguished by one single SNP in the target region.

The *C. suis*-specific real-time PCR is suitable for diagnosis in swine as successfully demonstrated in the present case report. Importantly, the *C. suis*-specific real-time PCR is also suitable for human samples, as it distinguishes *C. suis* from i) the human pathogens *C. trachomatis* and *C. pneumoniae* and ii) the currently known zoonotic species: *C. felis*, *C. abortus* and *C. psittaci*.

The detection of *C. suis* DNA as well as viable *C. suis* in the eyes of two slaughterhouse employees illustrates the value of this assay in future investigations on the zoonotic potential of *C. suis*. Becker et al. [41] showed intensively raised pigs to be pre-disposed to chlamydial associated conjunctivitis. Transmission from infected pig eyes to humans is perhaps not unusual in highly irritant environments like a slaughterhouse and intensive pig farms. *C. suis* positive persons had no clinical complaints and they showed no disease symptoms. We detected only low amounts of *C. suis* in the eyes, and perhaps *C. suis* only ended up in the eyes by touching face/eyes with contaminated hands. Possibly, it was just an eye “contaminant” and not a real infection. Perhaps serology would have clarified this issue. However, employees refused to give blood during our experiment. Thus, future studies in humans should include the detection of *C. suis*-specific antibodies in serum or at least in conjunctival swabs. None of the participating employees was infected with *C. trachomatis* and both *C. suis* isolates were tetracycline sensitive. Therefore, further research is needed, sampling larger human risk populations, to estimate the prevalence of *C. trachomatis* and Tc^R^ *C. suis* strains in people working with pigs. Besides, the potential of *C. suis* to cause pathology in the human eye needs to be examined in more detail, especially since *C. suis* is phylogenetically highly related to *C. trachomatis*, the etiology of human trachoma, and, as a recent study by Deborah Dean also found *C. suis* in the human eye [42].

In conclusion, we designed and validated a *C. suis*-specific real-time PCR for use in swine and humans. The assay can be used for detection and monitoring of *C. suis* strains in pigs and for examining their zoonotic potential more extensively. Additionally, the real-time PCR could be useful for preventing the spread of *C. suis* strains, for instance through international trade of boars and sperm.
